# Communication strategies for designing Facebook advertising campaigns to recruit rural participants to develop healthcare delivery interventions

**DOI:** 10.1017/cts.2020.5

**Published:** 2020-01-16

**Authors:** Elizabeth Flood-Grady, Deaven Hough, Rachel E. Damiani, Nioud Mulugeta Gebru, David A. Fedele, Robert F. Leeman, Janice L. Krieger

**Affiliations:** 1 STEM Translational Communication Center, University of Florida, Gainesville, FL, USA; 2 Clinical and Translational Science Institute, University of Florida, Gainesville, FL, USA; 3 Department of Advertising, College of Journalism and Communications, University of Florida, Gainesville, FL, USA; 4 Department of Health Education and Behavior, College of Health and Human Performance, University of Florida, Gainesville, FL, USA; 5 Department of Clinical and Health Psychology, College of Medicine, University of Florida, Gainesville, FL, USA; 6 Department of Psychiatry, Yale School of Medicine, New Haven, CT, USA; 7 Department of Health Outcomes and Biomedical Informatics, College of Medicine, University of Florida, Gainesville, FL, USA

**Keywords:** Communication, Facebook recruitment, rural health, healthcare delivery intervention development, elaboration likelihood model, message targeting

## Abstract

**Background:**

Little is known about designing research recruitment campaigns that connect with underserved, geographically isolated rural populations. A theoretically informed process is needed to assist research teams and practitioners in their evaluation of Facebook’s feasibility as a recruitment tool and development of online materials for recruiting rural adults into healthcare delivery intervention development studies.

**Methods:**

We drew from research and theory in communication and incorporated process analysis techniques to develop replicable procedures for designing and evaluating Facebook campaigns for rural recruitment. We describe our process and illustrate using two case studies.

**Results:**

Campaigns received approximately 1000 link clicks from the target rural demographic and successfully enrolled participants using Facebook as a primary method of recruitment. The rural tobacco intervention development study received a total of 477 link clicks, cost only $155.80, and enrolled three (23%) of its 13 participants from Facebook. The rural mental health intervention development study received a total of 518 link clicks, cost only $233.28, and enrolled 178 participants.

**Conclusions:**

Our process yielded two successful recruitment campaigns. Facebook was an affordable and efficacious strategy for enrolling adults in behavioral research studies on tobacco and mental health. Future work should apply these theoretical techniques to additional study topics and evaluate specific message features associated with recruitment.

Social media channels, such as Facebook, offer billions of users the unique opportunity to access and exchange important health information [1,2]. Individuals living in rural areas, who have higher rates of a myriad of preventable diseases [3], and are as likely their urban counterparts to use the Internet [4], actively use social and digital media to obtain health information [5]. As a leading social networking site, Facebook can minimize social and physical distance often experienced by rural adults. Moreover, its expansive reach makes it particularly useful for disseminating opportunities to participate in intervention development studies – research that actively engages potential recipients of interventions in research development – to geographically isolated, underserved rural populations. Facebook provides online tools to identify prospective rural audiences to participate in research and gives researchers the unique advantage of simultaneously recruiting for a study and evaluating recruitment campaign efficacy (e.g., metric evaluation). This dual recruitment and monitoring approach can facilitate effective recruitment and adapt to rural populations to enroll them in healthcare delivery intervention development studies.

However, challenges communicating with rural audiences about research participation can impede recruitment. Many adults who reside in rural locations and adults with low health literacy find it difficult to comprehend – and may misinterpret – common language used to describe research participation. For instance, rural adults perceive the metaphor “randomization is like flipping a coin” as akin to gambling with one’s health, making them less likely to participate in research studies [6,7]. In addition, rural audiences emphasize independence and self-sufficiency in their definitions of health, and their overall health attitudes and beliefs differ from adults living in urban areas [8]. Thus, the messages and information commonly used to communicate with individuals about health research opportunities may not capture the attention of rural audiences and may adversely affect participation.

Developing advertising campaigns that communicate messages that resonate with rural audiences is critical to engaging and recruiting underserved, rural populations into healthcare delivery intervention development studies. Leveraging Facebook’s expansive reach to disseminate research opportunities to rural audiences has the potential to reduce discrepancies in clinical enrollment between rural and urban audiences [9]. However, it remains unclear how to design and evaluate Facebook recruitment campaigns that connect with underserved, rural populations. Drawing from research and theory in communication, we describe a process – that worked for our institution – for evaluating the feasibility of Facebook as a study recruitment tool and developing theoretically informed materials for recruiting rural audiences into healthcare delivery intervention development studies. We use two case studies on rural Facebook recruitment to describe and illustrate this process.

## Methods and Procedures for Developing Facebook Advertising Campaigns for Rural Recruitment

Despite Facebook’s potential to revolutionize study recruitment, the tangible steps for designing, implementing, and evaluating Facebook research recruitment campaigns are unclear. Our theoretically informed process for engaging rural individuals in research studies through Facebook recruitment is contextualized, in part, through an explanation of the recruitment services provided by our institution’s Clinical Translational Science Institute (CTSI) Recruitment Center and two case studies wherein interdisciplinary teams used Facebook advertising to recruit rural audiences into healthcare delivery intervention development studies.

## CTSI Recruitment Center Consultations

Research teams who are interested in recruitment services, including Facebook advertising, complete an online form through the CTSI Recruitment Center website describing the nature of their study and recruitment needs. The form is emailed directly to the Recruitment Specialist (the center’s RS), who assesses the study needs and schedules an initial recruitment consultation with the team. Prior to the consultation, research teams may be asked to provide additional study information (e.g., eligibility criteria, previous recruitment efforts). During the initial consultation, the center’s RS provides research teams with an overview of the suite of available recruitment services, as teams may be unaware of the resources available at the university or have uncertainties trying new recruitment methods. Services include a consent to recontact registry, a community-based registry, a university-approved social media channel, and access to national research registries. Research teams are counseled as to which recruitment services are the best match for their study. If a team is interested in using Facebook recruitment, they receive an overview of Facebook advertising (e.g., how it works) and our institutional account (i.e., University of Florida (UF) Studies), which is led and managed by the CTSI Recruitment Center.

## Step 1: Assessing Facebook Recruitment Feasibility

To determine Facebook’s potential as a recruitment tool for a particular study, the center’s RS conducts a feasibility assessment. Feasibility assessments evaluate the potential of conducting a study in a specific location – given a particular population – and successfully completing the project in terms of timelines, prospective targets, and cost [10]. To determine Facebook recruitment feasibility the center’s RS completes a mock query for the study. The mock query identifies the number of prospective participants who are active users on the site, who fit the selected targeting criteria for the study, and who may ultimately see [11] and potentially engage with the advertisements (i.e., reach) in the geographic area where the study is conducted.

Prospective participants (i.e., active users on Facebook) for research recruitment are identified based on their demographics and interests. Targeting by demographics includes identifying users by age or age groups (e.g., adults age 65+, 18–44 years of age), gender (e.g., men, women), and lifestyle information as indicated by users personal Facebook account, such as relationship status (e.g., married). Demographics also include targeting individuals by their geographic location. For UF studies, we typically set campaigns to appear within a 50-mile radius of the city/location in which the study is being conducted (often Gainesville, Florida, the location of our main campus). The 50-mile radius encompasses a number of rural areas. We use demographic and location targeting in mock queries to determine the potential reach and link clicks – an estimate for the number of clicks the advertisement will receive on a daily basis [11], for a given study.

Interest targeting is crucial to identifying and recruiting individuals into health research and to determining feasibility. Interest targeting permits research teams to use key words on Facebook and users’ involvement in certain groups or organizations (e.g., as denoted by liking certain pages, involvement in groups) to further identify audiences within the previously set demographic criteria. For instance, interests such as “AARP” and “DIY Everywhere” could be used in a mock query to evaluate the reach for a study on adults ages 50 to 65+. Interests targeting also uses key words that relate to a specific disease, health topic, or condition, such cancer, diabetes, and nutrition, to identify users by their health interests. The center’s RS may use the Audience Insights tool from Facebook [12] to identify interests for a wider range of targeting criteria. This tool may be helpful for identifying and recruiting hard to reach populations or individuals whose interests are not easily identified, such as healthy volunteers. These interests may include local grocery store brands, sports teams, and restaurants. Multiple interests are included to accommodate for the potential of low reach and low link clicks.

Ad placement is also specified during the mock query. Per our institutional guidelines for using social media in research recruitment [13], ads can appear in user’s right-hand column and main newsfeed. Facebook’s right-column advertisements appear exclusively on desktop computers, whereas advertising that appears in Facebook users’ main newsfeeds can be viewed from desktop computers and mobile phones. Because advertising placement influences potential reach, it is essential to include in the mock query.

Facebook recruitment feasibility is largely determined by the potential reach and estimated link clicks for a particular study, based on the targeting parameters, and ad placement. Previous studies that have used the UF Studies for recruitment also used as a benchmark to determine if a study should proceed with Facebook advertising.

## Recruitment Case Studies

### Rural Tobacco Intervention Development Study, Case Study 1

The goal of this study was to explore rural tobacco users’ perceived barriers and motivators to participating in research and smoking cessation and use the qualitative findings to connect researchers with rural tobacco users. Individuals who were over the age of 18 years old, current tobacco users (i.e., used tobacco products, including cigarettes, electronic-cigarettes (e-cigs or vapes), and dry tobacco (e.g., snus and dip) on some days or everyday), and lived in a rural county in Florida were eligible to participate in the interview study. The team initially implemented “on the ground” recruitment approaches by utilizing their campus network to establish contact with citizen scientists, representatives from local government extension offices, and county department heads in the four rural counties where they were recruiting. Individuals helped the team identify locations and events (e.g., Amvets Sunday Funday) for recruitment. Limited success with this initial recruitment approach led the study team to work with the CTSI Recruitment Center to implement additional recruitment strategies, including i2b2 queries to identify prospective participants and Facebook paid advertising to recruit for the study. Individuals who participated in the study were remunerated with a $25 Walmart gift card.

### Rural Mental Health Communication Intervention Development Study, Case Study 2

The goal of this study was to identify and recruit rural parents with at least one adolescent to participate in an online survey about parent–child mental health communication and to provide feedback on existing web-based mental health resources. The data collected in this study served as the foundation for a larger pilot project aimed at engaging rural parents and adolescents to participate in the development of a web-based mental health intervention program for rural youth. Participants who were over the age of 18, lived in a rural county in Florida, and had at least one adolescent (i.e., a child between 10 and 17 years of age) were eligible to participate in the study and were recruited through Facebook paid advertising. Individuals who participated in the study were remunerated with $10 e-gift card.

### Rural Facebook Recruitment Feasibility

Study demographics (e.g., age, gender) and strategies that are specific to identifying and recruiting rural audiences for a particular study, such as rural zip codes, are used to determine rural Facebook recruitment feasibility. Rural audiences are also identified and targeted by study-related interests and interests that may resonate with people who reside in rural areas, as described below.

### Rural Facebook Recruitment Feasibility, Case Study 1

We used several strategies to evaluate the feasibility of Facebook for recruiting rural adults to participate in the tailored tobacco intervention development study. First, individuals were identified by their age and gender. Second, zip codes were used to identify and target individuals living in the north Florida rural counties of Bradford, Columbia, Levy, and Union. For this study, a county was considered “rural” if it was classified as rural by the Florida Department of Health (i.e., the county has 100 persons or less per square mile), the United States Department of Agriculture (USDA) (i.e., county is nonmetro-urban with a population of 2500–19,999, adjacent to a metro area or Nonmetro-Urban with a population of 20,000 or more, adjacent to a metro area), the Centers for Disease Control and Prevention (i.e., county is classified as micropolitan, with a population of 10,000–49,999 or noncore – counties are considered nonmetropolitan and do not meet criteria for micropolitan), and our institution’s classification (i.e., county is classified as small town/rural). Thus, rural recruitment feasibility was determined, in part, as Facebook users living in north Florida within a 50-mile radius of the cities/zip codes in the four rural counties.

Second, interests and key words pertaining to the study were included to assess feasibility. A total of 13 Facebook interests related to tobacco use, such as cigar, smoking, and vapor, were added to the query to identify prospective participants and determine reach (see Table 1). The Facebook “Audience Insights” tool provided a list of interests deemed relevant to rural adults living in the north Florida geographic region (e.g., Walmart, Tim Tebow, Universal Orlando, Publix), though these were not used to target individuals for this study (see Table 1 for full targeting criteria). Using these demographic and interest criteria, the potential reach for this campaign was 19,000 Facebook users.

**Table 1.** Targeting criteria and examples of language considerations for designing recruitment campaigns for rural audiences

	Facebook targeting criteria		Message considerations			
	Demographics	Interests	Identity	Credibility	Intrinsic and extrinsic appeals	Participation and illness framing
Rural tobacco intervention development study, Case Study 1	Men and women; adults over the age of 18; 50-mile radius of four rural counties in Florida	Camel (cigarette), cigar, cigarette, cigarette pack, electronic cigarette, Marlboro (cigarette), nicotine gum, smoking, snuff (tobacco), tobacco, tobacco pie, vapor, vaporwave	Rural; rural communities; Floridians who live in rural communities; Participants who live in rural Florida; North Florida rural communities	Researchers at UF; UF tobacco study; Rural tobacco study	You can contribute to research	The importance of participant’s unique input and individual experiences: Researchers are seeking input from Floridians who live in rural communities and who use tobacco products; You can help researchers at UF understand how rural Floridians use tobacco products.
					Compensation provided	
Rural mental health communication intervention development study, Case Study 2	Men and women; adults over the age of 18; 50-mile radius of rural counties in Florida (full list of rural counties available on request)	Parents with adult children (18–26 years), Parents with preteens (8–12 years), Parents with teenagers (13–18 years)	Parents; Parents of adolescents; Florida Parents of adolescents	Researchers at UF	Opportunity for Florida parents; Florida parents have the opportunity to play an important role in mental health research	Parents as important caregivers whose individual perspectives were central to understanding mental health: Parents of adolescents are invited to participate in an online survey about social and behavioral health
					e-gift card provided	Mental illness stigma: Communication about mental health and illness; online survey about social and behavioral health; mental health research

UF, University of Florida.

### Rural Facebook Recruitment Feasibility, Case Study 2

Similar strategies described in Case Study 1 were used to evaluate the feasibility of Facebook for recruiting rural parents of adolescents into the mental health communication intervention development study. First, individuals were targeted by their age and gender. Second, zip codes were also used to identify and target individuals living in rural counties across the state of Florida. For this project, a county was considered “rural” if it was classified as rural by the 2010 census (i.e., counties with 50,000 residents or less were considered rural). Thus, rural recruitment feasibility was determined, in part, as Facebook users living in the state of Florida who resided within a 50-mile radius of the cities/zip codes in the 31 rural counties.

Second, interests and key words pertaining to the study were included to assess feasibility. A total of three Facebook interests pertaining to parental status, including “parents with adult children (18–26 years),” “parents with preteens (8–12 years),” and “parents with teenagers (13–18 years),” were added to the query to identify prospective participants and determine reach (see Table 1 for full targeting criteria). Because our goal was to understand how – if at all – parents of adolescents communicate with their child about mental health and to receive feedback on existing web-based mental health resources, individuals were not identified and targeted using key words and interests pertaining to mental health. Linguistic considerations pertaining to mental health are described in the section on Developing Content for Facebook Advertising. Using these demographic and interest criteria, the potential reach for this campaign was 2,600,000 Facebook users.

### Additional Steps and Considerations

Research teams complete a recruitment walkthrough with the center’s RS using Facebook’s Ads Manager [14] business tool. This tool is the interface where the center’s RS selects and sets the criteria for identifying, targeting prospective participants, launches recruitment advertisements, and monitors Facebook advertising campaign progress (i.e., budget, clicks, reach, and impressions). A recruitment walkthrough via the Ads Manager provides a visual for how campaigns work, allows teams to see examples of previous study campaigns, including the target audience, campaign results, and budget, and demonstrates how the center’s RS monitors and reports ad metrics. Research teams only have access to the Ads Manager – and previous campaigns – when they complete the recruitment walkthrough with the center’s RS. Completing a recruitment walkthrough is critical to demonstrating the effort involved in developing, managing, and monitoring the recruitment campaigns, especially since the next item discussed is service fee.

The center’s RS reviews and confirms the fees associated with Facebook advertising (e.g., campaign cost) and Recruitment Center services (e.g., time and cost to developing ads, managing campaign progress). Research teams are advised to budget $250 a month for recruitment advertising on Facebook, though teams may commit to spending as much or as little they are comfortable with. Recruitment Center fees are not adjusted or waived based on campaign costs. Research teams receive a post-consultation email with notes from the consultation, next steps, and if applicable, details on Facebook recruitment feasibility.

## Step 2: Designing Recruitment Materials for Facebook Advertising Campaigns

If Facebook is deemed an appropriate channel for study recruitment, and the study team agrees to the costs, teams collaborate with the CTSI Recruitment Center on a comprehensive Facebook recruitment plan for submission and approval from the Institutional Review Board (IRB). Using standardized templates developed as part of the larger initiative at the university to establish procedures surrounding the use of social media in research recruitment, the plan includes a description of the UF Studies Facebook account, how the recruitment campaign will managed by the CTSI Recruitment Center [13], the targeting criteria used to determine feasibility, including additional targeting criteria identified by the center’s RS after the consultation. The content of recruitment materials, including the ad text, headlines, and images that may be used in advertisements (see Table 2 for descriptions).

**Table 2.** Key areas and descriptions included in the Facebook recruitment plan

Content Area	Description
Campaign overview	Description of the UF Studies Facebook account, how the page is managed by the CTSI Recruitment Center, and ad placement (i.e., where the ads will be seen by participants).
Targeting criteria	Options for targeting prospective participants including demographics, interests, and location (such as city, general area, or specific zip codes).
Adverting content	
Ad text	Summary of the study highlighting a diverse selection of key words describing identity features of the target population, calls to action and steps to participation, study credibility, and intrinsic and extrinsic appeals, in a maximum of 125 characters.
Ad Headlines	Concise description of the study, highlighting the study population, topic of the study, and institutional credibility in a maximum of 25 characters.
Ad photos	Diverse selection of at least six Shutterstock photos. Shutterstock photos are included in the Facebook advertising account and in the cost the Recruitment Center charges to investigators.

CTSI, Clinical Translational Science Institute; UF, University of Florida.

Investigators receive assistance creating theoretically informed recruitment messages. The center’s RS creates study recruitment plans that can be used for several ad sets, developing a minimum of six variations of text, headlines, and images for each study campaign. Including a variety of text and visual options increases the likelihood that advertising text will adhere to regulatory specifications used by IRBs to approve materials and ensures teams have enough content to continue running recruitment campaigns for several months. Because Facebook uses an algorithm to approve recruitment advertisements, developing multiple ad sets also accounts for potential issues that may arise with approval on social media. Developing content for multiple ad sets also provides our Recruitment Center with enough content to evaluate high- and low-performing ads and ensures the team has additional IRB-approved options if performance is low on a particular ad (i.e., if there are low link clicks). Using the standardized templates, research teams are responsible for submitting the CTSI Facebook recruitment plan with materials to the IRB for approval, making additional edits to the materials as needed, and submitting final copies of recruitment advertisements to IRB.

### Theoretical Considerations for Developing Facebook Recruitment Materials

There are important theoretical and practical considerations to designing effective recruitment advertisements on Facebook. The elaboration likelihood model (ELM) contends that audiences attend to messages actively or passively and (in)attention to message content is influenced by participant’s involvement with the message topic [15]. Audiences who perceive messages as personally relevant actively seek out and process this information [16,17], whereas uninvolved audiences (i.e., those who have little involvement or interest in a topic) passively attend to the same information with little awareness, comprehension, or evaluation [15,17]. Thus, a primary goal of designing Facebook recruitment materials is to highlight the relevance of the study and participation to prompt active processing and message engagement among active and passive members of the target audience.

### Identity Roles

It is important for messages to resonate with the identities and social group membership of the target audience [18]. To ensure messages resonate with intended audiences on Facebook, ads should be customized and incorporate language and visuals that align with multiple aspects of the target audience’s identity. Primary identities often stem from people’s membership in large social groups, such as ethnic [19] and age identities [20]. These identities often apply, more broadly, to larger social groups. Secondary identities reflect people’s identification with certain behaviors or with groups associated with a set of behaviors [21], such as identifying with exercising and healthy living. Tertiary identities are unique to the individuals being targeted in advertisements and reflect the extent to which people identify with their position in the social group and the labels used to describe membership affiliation [21], such as being a cancer survivor, parent of an adolescent, or a local sports fan.

Understanding and highlighting target audience identities are important to developing recruitment materials. When designing ads, we include as much about the study population and highlight identities that may be salient to target audiences. For instance, rather than describing a research opportunity as “A study seeking adult participants,” advertisements would highlight the primary identity characteristics of the intended target audience, such as their age (e.g., adults 65+) and tertiary identity roles (e.g., caregivers). A recruitment advertisement describing that same opportunity as “Adult caregivers between the ages of 65–80 needed to participate in a study” should increase message relevance among prospective participants.

Ad images emphasize prospective participants’ primary, secondary, or tertiary identities. For instance, if recruiting older adults (e.g., adults 55+) into a study on physical, images should capture older individuals from diverse backgrounds (e.g., women, men; African Americans, Latinx). Images can also demonstrate prospective participants enacting secondary identities relevant to the study (e.g., engaging in physical activity, such as walking or swimming). Healthy volunteer studies and those with broad eligibility criteria should incorporate images with individuals of all ages, races, and genders.

### Additional Theoretical Considerations

For advertising messages, different information and appeals may be effective for different audiences [15]. According to ELM, attitudes of audience members with low involvement with an advertisement are positively influenced by appeals not directly related to the ad topic, such as the attractiveness, credibility, and structural features of the ad [15]. In other words, members of the Facebook target audience who were identified using interests and key words but may have little interest in participating healthcare delivery intervention development studies are likely to engage the ads because of information not directly related to the study. To account for these individuals, we consider additional message features when designing Facebook recruitment materials, including credibility, intrinsic and extrinsic appeals, calls to action (CTAs), and diversity. Features are reflected in both the content (information presented in the text and headlines) and visual (photos) aspects of materials.

#### Credibility

This refers to the trustworthiness of the source [22] communicating a message. Medical experts are widely considered as credible sources of health information [23] and may be perceived as credible sources of information about recruitment. Variability in perceptions of source credibility across social groups [15,24] suggests that how the source is presented visually (e.g., doctor vs. lay person; female vs. male; African American vs. Caucasian) and linguistically in messages (e.g., student with background in nutrition vs. medical research institution), which may influence message engagement and recruitment. Credibility is presented in multiple ways to account for variability in audience perceptions, including an emphasis on the institution (e.g., “University of (name)”) and/or researcher conducting the study (e.g., “Researchers at (institution)”). If applicable, ads may also demonstrate a personal connection between the researcher and target audience.

#### Intrinsic and extrinsic appeals

A recent study found that medical research institutions communicate with prospective participants about research online using intrinsic (i.e., internal factors; e.g., interest in advancing science, helping a loved one) and extrinsic (i.e., external factors; e.g., time, transportation, compensation) message appeals [25]. Institutions emphasized intrinsic nonmonetary motivations for participating in research, such as the desire to advance science and to help oneself [25] on their websites. Extrinsic appeals, such as study incentives, were also described on institutions’ websites. Thus, intrinsic and extrinsic appeals are included in recruitment messages. Nonmonetary motivations highlighting internal factors for participating are included – and vary – across ad text (e.g., “By participating, you can help advance the future of science.”). Extrinsic factors that could encourage participation, such as offering compensation (e.g., “You will receive an e-gift card”) or highlighting the time needed to participate (e.g., “Participating will take 30 min”), are also included – and vary – across ads.

#### Calls to action

Calls to action (CTAs) encourage specific behavior among the individual seeing the ad and are reflected in the ads by describing active steps individuals can take to learn more about the study (e.g., “Click here to access the study website”) or to participate (e.g., “Click here to be directed to the survey”). CTAs may motivate members of a target audience who do not identify with the individual, group, or behaviors depicted in ad to interact with or engage in additional behaviors pertaining to the study.

#### Diversity

Clinical research routinely fails to adequately enroll minority and underserved populations into studies [26]. Thus, diversity is among the most important considerations for designing Facebook advertisements. Diversity is reflected in the images selected and language used to describe research studies and activities in the recruitment advertisements. In the content, we use numerous words and phrases to describe a study and identities of participants, to highlight credibility, to explain intrinsic and extrinsic appeals, and CTAs. Diversity is reflected in images by representing individuals of as many ages, races, and genders as possible – as they fit with the study population. See Table 3 for descriptions of themes and examples.

**Table 3.** Themes, descriptions, and examples of theoretical considerations included in recruitment materials

Theme	Description	Examples
Identity	Reflect individual’s membership in various social groups through an explanation of their primary, secondary, and tertiary group memberships.	Age (e.g., adults 65+), race (e.g., African Americans, Hispanics), gender (e.g., women, men, nonbinary) (*primary*)
		For a physical activity study, language describing types of physical activity (e.g., walking, running) and images demonstrating individuals engaging in physical activity (*secondary*)
		“Parents, Guardians”; “Caregivers, Patients”; “Teens, adolescents”; “Smokers “vs. “Tobacco users” (*tertiary*)
Credibility	Describe the trustworthiness of the source communicating a message. The credibility of the institution and/or the researcher conducting the study is emphasized in recruitment messages.	“Medical Researchers”; “Researchers at (name) institution”; “Researcher who has personal experience with adoption”; “Physician researchers”
Calls to Action	Encourage specific action on the part of the individual seeing the ad by describing steps individuals can take to learn about the study or to participate.	“Click on the link to see if you qualify”; “Click here to be directed to the survey”; “Click here to access the study website”; “Learn more”
Intrinsic and extrinsic appeals	Reference internal factors (e.g., interest in advancing science) and external factors (e.g., time, transportation, compensation) that may motivate participation.	“You have the power to help us fight obesity!”; “By participating, you can help advance the future of science”; “By participating, you can help the health of your community” (i*ntrinsic*)
		“Compensation provided”; “Participating will take 30 min” (*extrinsic*)

*Note*. Diversity is reflected in the language used to describe studies in recruitment materials, including numerous words and phrases to describe the study and identities of participants, to highlight credibility, to explain intrinsic and extrinsic appeals and calls to action. Diversity is reflected in images by representing individuals of as many ages, races, and genders as possible.

### Advertising Text

Also described as “ad or post text,” advertising text refers to the content presented in the body of the Facebook advertisement. Identities (e.g., “Smokers,” “parents”), credibility (e.g., “Medical researchers”), intrinsic and extrinsic appeals (e.g., compensations provided”), and CTAs (e.g., “Learn more”) are included in the text of advertisements (see Table 3 for additional examples). Ad text must also adhere to regulatory, institutional guidelines and Facebook guidelines. For instance, Facebook permits up to 125 characters in the post text. Similar to offline advertisements (e.g., flyers), Facebook advertisements cannot target audiences by health or illness conditions. Language used in high-performing ads (e.g., ads with high clicks) and how words or phrases may be perceived by the target audience are also considered when developing text of recruitment materials.

### Advertising Headlines

With a 25-character limit for ad headlines, it is important to succinctly summarize the study for participants and for mobile optimization. Similar to the post text, information about the target population and specific aspects of the study are included in the headlines. Due to space constraints, we choose key words and interests that best reflect the study and represent the identities of prospective participants. For instance, if recruiting men who meet men online to participate in a study testing online dating applications, we would include the primary (e.g., men) or tertiary group identities (e.g., online daters) in the headlines. Credibility is also incorporated into the headline, often by using a common abbreviation for our institution and health system (see Table 3 for additional examples). Intrinsic and extrinsic appeals are not included in headlines.

Advertisements are required to include a “learn more” button, which is embedded in the ad and directs users to a space outside of the social media site. Per our institutional guidelines, the “learn more” button directs prospective participants to a secure channel outside of Facebook, such as a URL for a university study, an IRB-approved website created by study team, or a Qualtrics or REDCap survey, for additional information about the study. Ad images function similarly to the “learn more” button and when clicked on, direct individuals to the separate, secure channel for study information.

### Images

Including images that reflect diverse populations and that represent the context of the study is crucial to recruitment. In addition to selecting images that represent the study population, we choose images that appear “realistic” (i.e., are not visibility or highly modified), represent diverse individuals (e.g., people who are non-White and White) in everyday settings (e.g., talking on the phone), and have performed well in previous UF Studies recruitment campaigns. Images are selected from the website Shutterstock, and the watermarked images are copied and pasted into the recruitment plan for submission and review from the IRB.

### Special Considerations for Designing Messages for Rural Audiences

Rural communities have unique needs, health concerns, demographic variation, and economic resources [27]. Like all cultural groups, rural communities are not monolithic. However, it is important for message designers to be aware that rural cultural values may be distinct from other populations. For example, research suggests that rural individuals are more likely to prescribe to traditional norms and conservative values [28]. In addition, rural individuals may experience health differently than non-rural populations [8]. Rural attitudes and beliefs about health emphasize independence, self-sufficiency, and ability to perform social roles and perceived obligations [8].

Rural audiences also hold distinct views of healthcare and caregiving. For instance, some rural adults are skeptical of outsiders and distrusting of medical professionals, in particular [29]. In rural communities, family members are also central to caregiving [30], and parents are crucial to teens and children’s ability to access care, especially in the context of mental health [27]. Incorporating the themes and identities consistent with rurality and rural audience’s health perceptions should help to facilitate recruitment of rural adults into health research studies. Limited trust in healthcare systems, coupled with an emphasis on family members as caregivers, further emphasizes the need to engage rural audiences in the participation and development of healthcare delivery interventions. Thus, highlighting rural identity, independence, and self-sufficiency and emphasizing other salient social identity roles of rural adults are crucial to designing recruitment messages for rural audiences.

### Developing Facebook Recruitment Materials for Rural Audiences, Case Study 1

To recruit rural adults into the tobacco tailored intervention development study, ads incorporated rural identity and framed study participation around independence and giving individuals agency to participate in their healthcare. Rural identity was incorporated into ads by using key words and phrases emphasizing rurality, such as “rural” and “Floridians who live in rural communities.” Participation in the study was framed around independence and ability, and we used language that conveyed the importance of participant input and individual experiences in helping researchers understand their health decisions (e.g., “You can help researchers at UF understand how rural Floridians use tobacco products”).

The institution and researchers were highlighted as sources of credibility (e.g., “UF tobacco study”), intrinsic (i.e., internal factors, e.g., the desire to help oneself or to advance science) and extrinsic (i.e., external factors, e.g., “compensation provided”) messages, and CTAs (e.g., “Click here to visit the study website”) were also included to increase message appeal. Key words reflecting the content of the study, such as tobacco, tobacco products, and tobacco use, were also incorporated into recruitment advertisements (see Table 4 for examples of rural language considerations).

**Table 4.** Metrics for rural Facebook recruitment campaigns

	Total potential reach	Actual reach	Link clicks	Enrollment	Campaign cost
Rural tobacco intervention development study, Case Study 1	16,000	4940	477	13	$155.80
Rural mental health communication intervention development study, Case Study 2	2,600,000	12,536	518	178	$238.28

*Note*. Enrollment for Case Study 1 reflects the total participants enrolled in the study, which includes the three participants who were enrolled from Facebook.

Rural settings are not culturally homogenous [8]. Thus, it was important to select photos with diverse individuals and images of people in rural settings. Images with individuals looking at the camera were selected for this recruitment plan. These types of images performed well in previous campaigns and reinforced cultural values of independence. Photos depicted individuals whose demographics were consistent with individuals living in the four North Florida counties (e.g., photos included individuals from African American, White, non-White, Hispanic backgrounds). Photos with individuals in green, outdoor spaces were selected to represent rurality, whereas city settings with skyscrapers and landscapes that were dissimilar to North Central Florida (e.g., a desert, beach) were excluded.

### Developing Facebook Recruitment Materials for Rural Audiences, Case Study 2

To recruit parents of adolescents into the mental health communication intervention development study, ads highlighted the role of parental identity and framed participation in ways that offered parent participants agency to inform the development of future healthcare efforts for their children. Tertiary identities were incorporated into ads by using key words and phrases emphasizing parental identity roles, such as “Parents of adolescents.” Participation was framed around parental identity and caregiving; ad language^1^ positioned parents as important caregivers whose individual perspectives were central to understanding mental health (e.g., “Researchers want to understand parent adolescent communication about mental health and illness”).

The institution and researchers were highlighted as sources of credibility (e.g., “Researchers at UF”). Ads also emphasized parental credibility, intrinsic (e.g., “Opportunity for Florida parents”) and extrinsic appeals (e.g., “e-gift card provided”), and CTAs (e.g., “Click on the link to see if you qualify”) were also included to increase message appeal. Images depicting a diverse selection of parents and adolescents – talking and engaging with technology – were selected for the recruitment plan as parents of adolescents were the target audience of this study. Thus, images demonstrated prospective participants enacting parental identities.

We also considered the potential for mental illness stigma to influence recruitment. Mental illness stigma – public, perceived, and self – is a significant barrier to rural participation in mental health-related services (e.g., treatment, research, etc.) [31]. To account for the stigma that could preclude or deter parental participation, mental illness communication was framed in multiple ways (e.g., communication about social and behavioral health) (see Table 4).

## Step 3: Tracking Facebook Advertising Campaigns and Study Recruitment

Tracking Facebook study recruitment is a joint effort between the center’s RS and the research teams. The center’s RS sends teams weekly updates documenting campaign progress (i.e., total ad clicks, reach, comments), remaining budget, and how many weeks remain in the campaign. Screenshots of user comments are also included in weekly updates, as necessary^2^. This regular monitoring allows the center’s RS and teams to make adjustments, as needed, to maximize recruitment. At the end of the campaign, teams also receive a final summary with their total metrics and suggestions for future campaigns.

To track the efficacy of Facebook recruitment, research teams include an item in their survey, in-take or screener, asking participants how they heard about the study, and include Facebook as a response option. Research teams are also expected to respond to weekly updates with data on the number of participants who inquired about the study/filled out a screening survey and enrollment. Inquiries are defined as phone calls and emails from potential participants to the study team. Completion of online screeners (for surveys) is also considered inquiries. Enrollment is the number of individuals who passed screening, consented, and participated in the study. Teams are aware that they will be asked to report on metrics before the campaign begins. Because participants may continue to inquire about the study after campaigns have ended, research teams also update the center’s RS on inquiries and enrollment after the study is officially closed. As an incentive for reporting timely recruitment data, researchers may be invited to participate in future manuscripts on recruitment.

## Results

### Facebook Advertising Campaign, Case Study 1

**Fig. 1.**
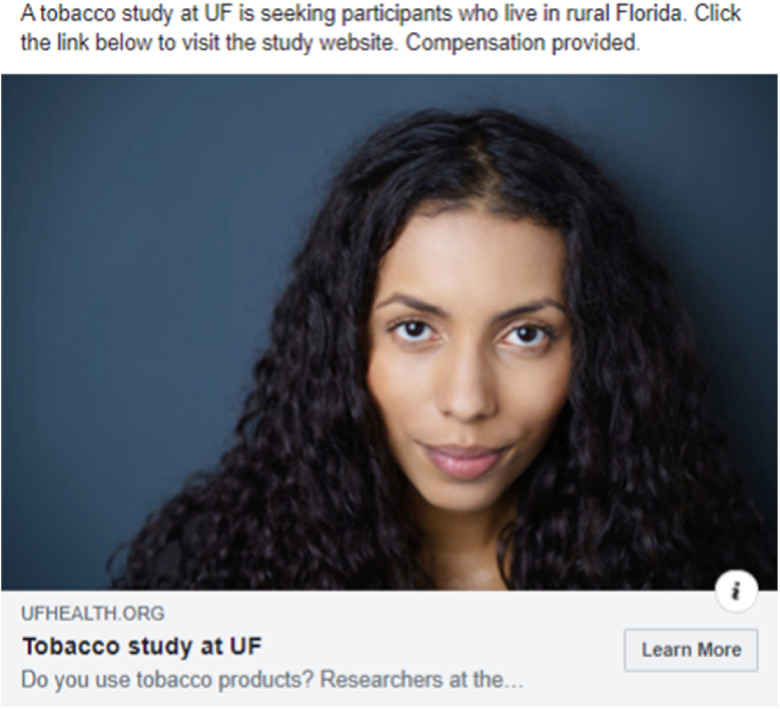
Highest performing ad from Case Study 1.

A total of 12 ads (2 ad sets) were disseminated over three weeks. This campaign received a total of 477 link clicks and cost $155.80. The highest performing ad received 233 link clicks (see Fig. 1). Sixteen individuals who saw the advertisements on Facebook inquired about the study, three of which enrolled (see Table 4 for full metrics).

UF, University of Florida.

### Facebook Advertising Campaign, Case Study 2

**Fig. 2.**
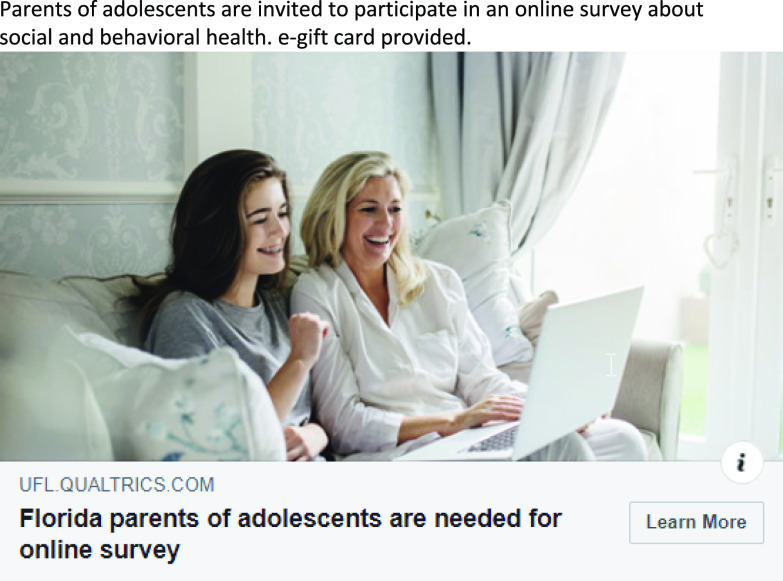
Highest performing ad from Case Study 2.

A total of six ads (one ad set) were disseminated over four weeks. This campaign received a total of 518 link clicks and cost $233.28. The highest performing ad received 340 link clicks (See Fig. 2). Four hundred and thirty-five participants inquired about the study 178 of which enrolled (See Table 4 for full metrics).

## Discussion

### Evaluating the Recruitment Process

In this paper, we described a theoretically informed process for evaluating Facebook’s feasibility as a study recruitment tool and developing materials for recruiting rural adults to participate in the development of healthcare delivery interventions. Engaging multiple theoretical strategies to develop recruitment advertisements for dissemination on Facebook that target – and appeal to – prospective participants is important to prompting active processing and message engagement among active and passive members of a target audience. Using Facebook’s online tools to select and set criteria to target prospective participants for study advertisements were useful to determine the potential reach for the studies. However, incorporating message targeting strategies (i.e., identifying group level characteristics of a target audience (e.g., demographic, geographic, cultural, risk cognitive factors)) [32,33] and customizing the content of recruitment advertisements to address the identities, information needs, and preferences of prospective participants likely increased message relevance among prospective participants. Thus, rural recruitment success may be attributed to the dual-targeting approach to identifying rural audiences through the social media channel and strategically communicating with rural adults about research participation through carefully designed messages about recruitment.

## Lesson Learned, Recommendations, and Next Steps

We learned several important lessons as we developed Facebook advertising campaigns for rural recruitment and offer recommendations and next steps based on our experience. Some recommendations are specific to rural recruitment, whereas others apply more broadly to Facebook recruitment. First, metrics tracking through Facebook provides the Recruitment Center and teams with valuable information on recruitment. Through metrics tracking, teams learn which ads are high and low in terms of performance. Ad metrics also provide information regarding the language, text, and visual depictions that teams should include in future recruitment materials. Knowledge gleaned from metrics, specifically which language and visuals work best with the target population, can inform other aspects of the research design. For instance, if advertisements with individuals in informal clothing (e.g., jeans) perform better than those with individuals dressed to fit a certain role (e.g., doctor, researcher), when meeting with participants, teams can use this information to tailor their clothing to match the individuals depicted in the high-performing ads. Thus, as a communication channel, Facebook advertising facilitates study recruitment and the metrics offer insight regarding how to communicate and establish rapport with participants.

Second, linking Facebook advertisements to a landing page or website affiliated with the institution conducting the study may increase perceptions of study credibility. Health websites with certain structural features (e.g., organizational information, such as a physical address) are perceived as credible by online users [31,34]. Directing individuals to an institution-sponsored study listing page may enhance study credibility by presenting prospective participants study information on a page hosted by a reputable institution. Indeed, linking study advertisements to a secure survey screener (e.g., REDCap) can be effective at recruiting and screening prospective participants (the rural mental health study linked ads to a secure screener); however, online consent forms and screeners may be less likely to include the visual heuristic cues (e.g., institution logo) that may enhance study and team trustworthiness.

Third, we recommend running ad campaigns in one-month increments to establish a baseline for which images, text, and headlines work best with the target audience for use in campaigns. This timing also works well if teams need to “pause” or temporarily stop running ads (e.g., if a team receives more inquiries than they can reasonably manage) without major disruption. Fourth, consulting with investigators, designing theoretically informed recruitment messages, launching and monitoring campaigns, and tracking recruitment require a considerable amount of time. This process works best if teams collaborate with the center’s RS in advance of study deadlines and provide timely recruitment data. Because tracking recruitment can be difficult and time consuming, future efforts should explore options to integrate with existing data infrastructure at the institution to track recruitment and enrollment.

## Strengths, Limitations, and Conclusions

Our paper is limited by its descriptive evaluation of two Facebook recruitment campaigns. However, our process for developing theoretically informed recruitment materials for engaging rural individuals in healthcare delivery intervention development studies through social media is the first of its kind and a crucial step to understanding the efficacy of Facebook as a recruitment tool. Future studies should evaluate the themes presented in the content of Facebook recruitment messages to understand how ad themes relate to enrollment. This paper is also limited by its use of plain text and static images in recruitment ads. Future campaigns should include multiple message features (e.g., statistics, testimonials) and formats (e.g., videos, images) in recruitment campaigns and evaluate their efficacy in terms of metrics and enrollment. In sum, our theoretically informed process for developing recruitment materials and engaging rural individuals in healthcare delivery intervention development studies yielded two successful recruitment campaigns, and Facebook was an efficacious strategy for enrolling adults in research studies on tobacco and mental health.

## Footnotes

1 Although “rural” was inadvertently left out in the creation of the advertisements for Case Study 2, the campaign highlighted identities salient to recruiting rural adults into health studies and was successful in terms of recruitment.

2 If a potential participant commented that they were trying to reach the study team, the RS would take a screenshot of this comment and send it to the team to follow-up.
